# The InBIO Barcoding Initiative Database: DNA barcodes of Portuguese Diptera 01

**DOI:** 10.3897/BDJ.8.e49985

**Published:** 2020-03-20

**Authors:** Sonia A Ferreira, Rui Andrade, Ana R Gonçalves, Pedro Sousa, Joana Paupério, Nuno A Fonseca, Pedro Beja

**Affiliations:** 1 CIBIO/InBIO, Centro de Investigação em Biodiversidade e Recursos Genéticos, Universidade do Porto, Campus Agrário de Vairão, 4485-661 Vairão, Vila do Conde, Portugal CIBIO/InBIO, Centro de Investigação em Biodiversidade e Recursos Genéticos, Universidade do Porto, Campus Agrário de Vairão, 4485-661 Vairão Vila do Conde Portugal; 2 Rua Calouste Gulbenkian 237 4H3, 4050-145, Porto, Portugal Rua Calouste Gulbenkian 237 4H3, 4050-145 Porto Portugal; 3 Computational Biology and Population Genomics Group, cE3c, Faculdade de Ciências, Universidade de Lisboa, Campo Grande, 1749-016, Lisboa, Portugal Computational Biology and Population Genomics Group, cE3c, Faculdade de Ciências, Universidade de Lisboa, Campo Grande, 1749-016 Lisboa Portugal; 4 CIBIO/InBIO, Centro de Investigação em Biodiversidade e Recursos Genéticos, Instituto Superior de Agronomia, Universidade de Lisboa, Tapada da Ajuda, 1349-017, Lisboa, Portugal CIBIO/InBIO, Centro de Investigação em Biodiversidade e Recursos Genéticos, Instituto Superior de Agronomia, Universidade de Lisboa, Tapada da Ajuda, 1349-017 Lisboa Portugal

**Keywords:** Diptera, occurrence records, continental Portugal, DNA barcode, COI

## Abstract

**Background:**

The InBIO Barcoding Initiative (IBI) Diptera 01 dataset contains records of 203 specimens of Diptera. All specimens have been morphologically identified to species level, and belong to 154 species in total. The species represented in this dataset correspond to about 10% of continental Portugal dipteran species diversity. All specimens were collected north of the Tagus river in Portugal. Sampling took place from 2014 to 2018, and specimens are deposited in the IBI collection at CIBIO, Research Center in Biodiversity and Genetic Resources.

**New information:**

This dataset contributes to the knowledge on the DNA barcodes and distribution of 154 species of Diptera from Portugal and is the first of the planned IBI database public releases, which will make available genetic and distribution data for a series of taxa. All specimens have their DNA barcodes made publicly available in the Barcode of Life Data System (BOLD) online database and the distribution dataset can be freely accessed through the Global Biodiversity Information Facility (GBIF).

## Introduction

Diptera is one of the most diverse, abundant and widespread insect orders, with more than 158 000 described species, and many more still to be described (Pape et al. 2009; Evenhuis and Pape 2019). Dipterans are ubiquitous in many terrestrial ecosystems and larval stages of some species can also been found in aquatic ecosystems. They play important ecological roles in ecosystems, including those of pollinators, detritivores and parasites. Some species are also important disease vectors (Merritt et al. 2009) and crop pests (Skuhravá et al. 2010).

In continental Portugal, 1 475 species of Diptera were recorded, a sizable diversity, but small when compared to the 5 800 species known to occur in continental Spain (Carles-Tolrá 2002). Since the seminal work of Carles-Tolrá (2002), further increments to the Portuguese dipteran fauna have been made (*e.g. Andrade and Gonçalves 2014*; Ebejer and Andrade 2015; Pollet et al. 2019), but much remains to be known about its diversity and distribution patterns in the country. The huge diversity of this order, the shortage of specialised taxonomists and the difficulties in identifying many species are the main obstacles to overcome this lack of knowledge.

DNA barcoding is a method that aims to identify organisms based on a short DNA sequence previously sequenced from morphologically identified specimens (Hebert et al. 2003). This requires the construction of comprehensive reference collections of DNA sequences that represent the existing biodiversity (Baird et al. 2011, Kress et al. 2005, Ferreira et al. 2018). DNA barcoding can also be used as a first step in new species discovery and, as such, can be used as a tool to help address the taxonomic impediment problem (*e.g.* Kekkonen and Hebert 2014).

The striking scarcity of genetic data associated with the high biodiversity found in Portugal instigated the creation of a DNA barcoding initiative by the Research Network in Biodiversity and Evolutionary Biology - InBIO (Associate Laboratory). The InBIO Barcoding Initiative (IBI) makes use of Next Generation Sequencing technologies (NGS) to develop a reference collection of DNA barcoding sequences, focusing on Portuguese invertebrate taxa. Within the project, a special focus is afforded to insects, given their relevance to food webs and ecosystems functioning (e.g., Weisser and Siemann 2004; Mata et al. 2016; Silva et al. 2019). Furthermore, for many insect species occurring in Portugal, there are no barcodes available in public databases (Corley and Ferreira 2017; Corley et al. 2017; Weigand et al. 2019), and those that exist often show high distances to sequences obtained in Portugal, which may indicate cryptic diversity (Corley et al. 2019, Corley et al. 2019; Ferreira et al. 2018).

The IBI Diptera 01 dataset contains records of 203 specimens of Diptera collected in continental Portugal, all of which were morphologically identified to species level, for a total of 154 species. This is the first IBI dataset to be released to the Global Biodiversity Information Facility (GBIF) and all specimens have their DNA barcodes made publicly available in the Barcode of Life Data System (BOLD). We have included in this dataset the barcodes of all identified Diptera specimens in IBI up to December 2019, except those from the families Tipulidade and Limoniidae, for which we will provide a more detailed treatment in a future paper, due to the detection of new species and the need for further research. Overall, this paper is a contribution to sharing and publicly disseminating the distribution records and DNA barcodes of specimens from our reference collection to increase the available information on Portuguese Diptera fauna.

## General description

### Purpose

This dataset aims to provide a first contribution to an authoritative DNA barcode sequences library for Portuguese Diptera. Such a library should facilitate DNA-based identification of species for both traditional molecular studies and DNA-metabarcoding studies and constitute a valuable resource for taxonomic research on Portuguese Diptera and its distribution.

### Additional information

A total of 203 specimens of dipterans were collected and DNA barcoded (Suppl. material 2). Fig. 1 illustrates examples of the diversity of species that are part of the dataset of distribution data and DNA barcodes of Portuguese Diptera 01. All sequences of cytochrome c oxidase I (COI) DNA barcodes are 658 bp long. From the 154 species barcoded, twenty nine (19%) from 16 families are new to the DNA barcode database BOLD at the moment of the release (marked with * in Species field of Table 2). Forty-two additional species (27%) from 24 families were previously represented in BOLD, but with less than 10 DNA barcode sequences at the moment of the release (marked with '' in Species field of Table 2). Therefore, this dataset represents a significant contribution to enhance the species and genetic diversity of Diptera fauna represented in public libraries.

## Project description

### Title

The name “DNA barcodes of Portuguese Diptera 01” refers to the first data release of DNA barcodes and distribution data of dipterans within the InBIO Barcoding Initiative.

### Personnel

Pedro Beja (project coordinator), Nuno Fonseca (project chair), Sónia Ferreira (taxonomist and IBI manager), Joana Paupério (IBI manager), Pedro Sousa (project technician), all affiliated to CIBIO-InBIO, University of Porto; Rui Andrade (taxonomist) independent researcher and Ana Rita Gonçalves (taxonomist) cE3c, Faculty of Science, University of Lisbon.

### Study area description

**Figure 2.**
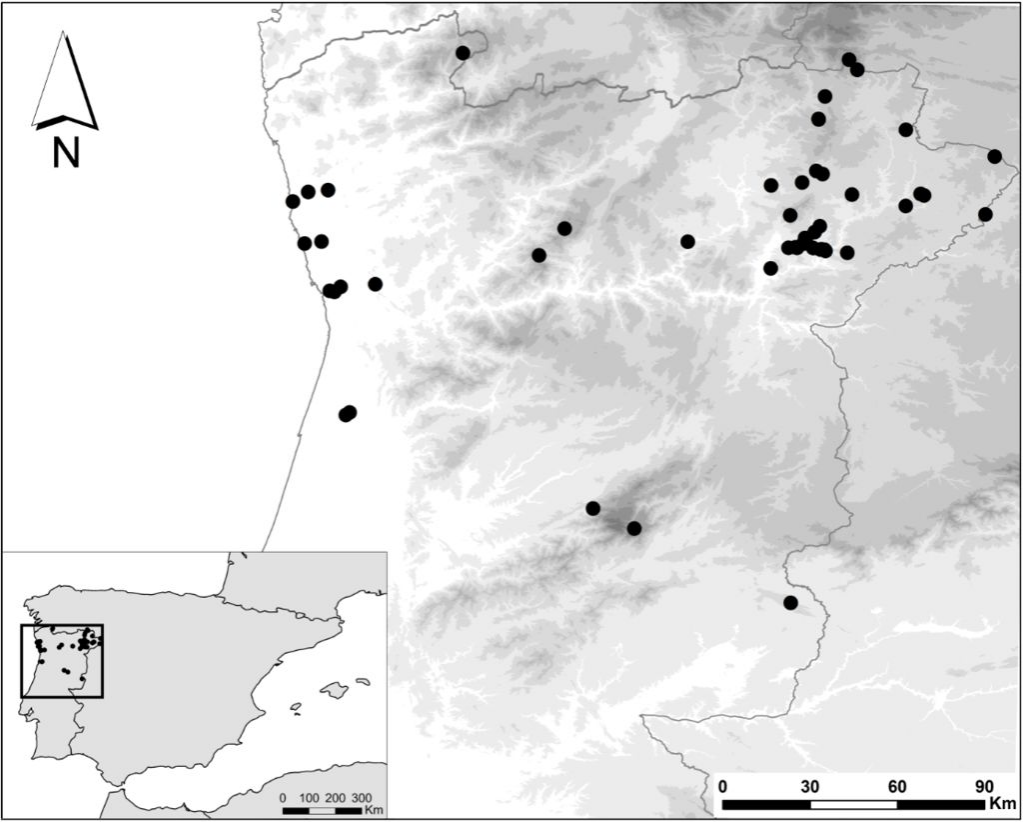
Map of the localities where Diptera samples were collected in northern Portugal.

North of the Tagus river in Portugal (Fig. 2).

### Design description

Dipteran specimens were collected in the field, morphologically identified and DNA barcoded.

### Funding

This project is funded by the European Union’s Horizon 2020 Research and Innovation programme under grant agreement No 668981 and by the project PORBIOTA—Portuguese E-Infrastructure for Information and Research on Biodiversity (POCI-01-0145-FEDER-022127), supported by Operational Thematic Program for Competitiveness and Internationalization (POCI), under the PORTUGAL 2020 Partnership Agreement, through the European Regional Development Fund (FEDER), by EDP Biodiversity Chair, and is part of research conducted at the Long Term Research Site of Baixo Sabor (LTER_EU_PT_002). P.S. was funded by the project ECOLIVES – Fostering sustainable management in Mediterranean olive farms: pest control services provided by wild species as incentives for biodiversity conservation (PTDC/AAG-REC/6480/2014/), supported by Portuguese national funds by FCT/MCTES and co-financed by Fundo Europeu de Desenvolvimento Regional (FEDER) throughout COMPETE – Programa Operacional Factores de Competitividade (POFC).

## Sampling methods

### Study extent

North of the Tagus river in Portugal

### Sampling description

The studied material was collected in 59 different localities from the northern half of continental Portugal (Fig. 2, Table 1). Sampling was conducted between 2014 and 2018 on a wide range of habitats, using mainly hand-held sweep-nets or direct search for specimens. Collected specimens were examined both dry and in alcohol using a binocular stereoscopic microscope (Optika ST-30-2LR, 20x-40x) and stored in 96% ethanol for downstream molecular analysis. Morphological identification was performed, based on keys and descriptions from literature (Suppl. material 1).

DNA extraction and sequencing followed the general pipeline used in the InBIO Barcoding Initiative. Briefly, genomic DNA was extracted from leg tissue using EasySpin Genomic DNA Tissue Kit (Citomed) following manufacturer’s protocol. The cytochrome c oxidase I (COI) barcoding fragment was amplified as two overlapping fragments (LC and BH), using two sets of primers: LCO1490 (Folmer et al. 1994) + Ill_C_R (Shokralla et al. 2015) and Ill_B_F Shokralla et al. 2015) + HCO2198 (Folmer et al. 1994), respectively. The COI mitochondrial gene (Folmer region), was then sequenced in a MiSeq benchtop system. OBITools (https://git.metabarcoding.org/obitools/obitools) was used to process the initial sequences which were then assembled into a single 658 bp fragment using Geneious 9.1.8. (https://www.geneious.com).

### Quality control

All DNA barcodes sequences were compared against the BOLD database and the 99 top hits were inspected in order to detect possible issues due to contaminations or misidentifications. Prior submission to GBIF, data was checked for errors and inconsistencies with OpenRefine 3.2 (http://openrefine.org).

### Step description

Specimens were collected in 59 different localities of continental Portugal. Sampling was conducted from 2014 to 2018, and consisted of direct search of specimens (e.g. *Hecamede albicans, Eutropha fulvifrons, Canace nasica*), the use of entomological nets to intercept specimens flight (e.g. *Hemipenthes morio*, *Sphaerophoria scripta*) or to sweep the vegetation (e.g. *Opetia nigra*, *Trigonometopus frontalis*). Specimens collected were stored in 96% ethanol. A tissue sample was removed, from which DNA was extracted and the COI DNA barcode fragment was sequenced. Data generated were submitted to BOLD, GenBank and GBIF.

## Geographic coverage

### Description

Continental Portugal

### Coordinates

40.054 and 42.002 Latitude; 6.274 and -8.774 Longitude.

## Taxonomic coverage

### Description

**Figure 3.**
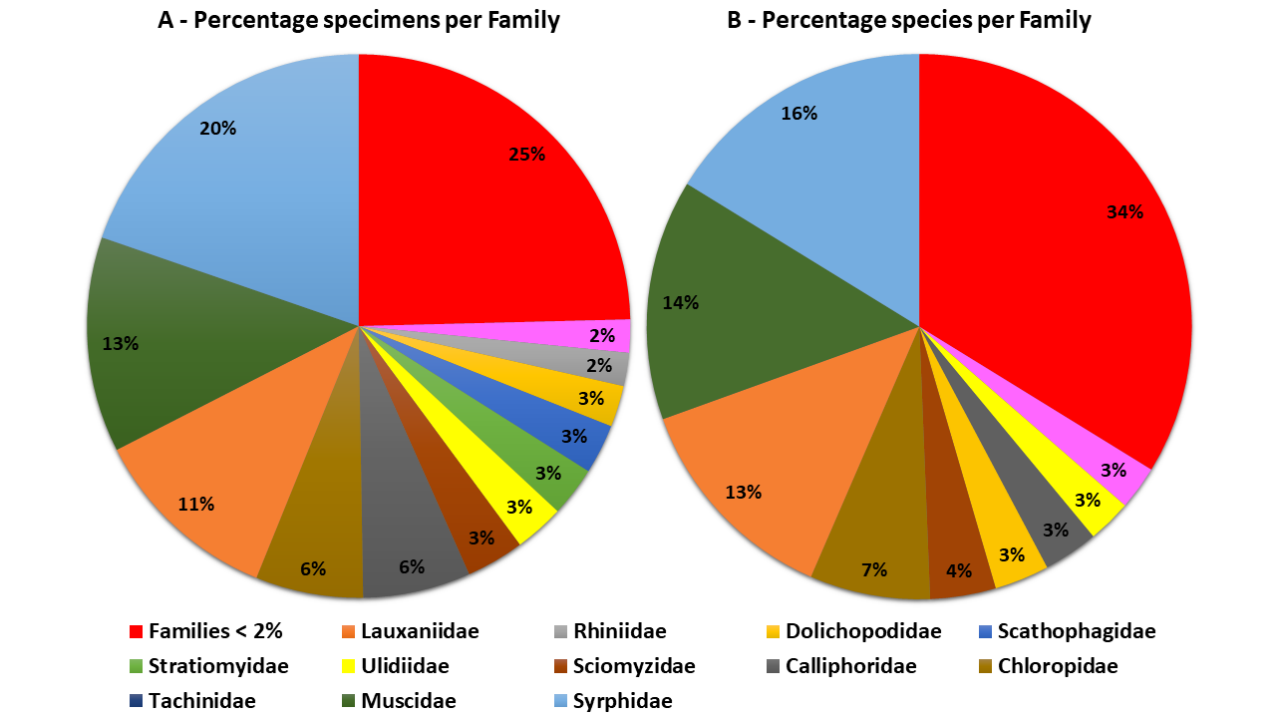
Distribution of specimens (A) and species (B), in percentage, per Diptera family present in the dataset. Families representing less than 2% of specimens/species were lumped together.

This dataset is composed of data relating to 203 Diptera specimens. All specimens were determined to species level. Overall, 154 species are represented in the dataset. These species belong to 41 families. Five families account for 56% of the total collected specimens, Syrphidae, Muscidae, Tachinidae, Calliphoridae and Chloropidae (Fig. 3). These five families account for 54% of the total species represented (Fig. 3). Nineteen families are represented by a single species.

## Temporal coverage

**Data range:** 2014-5-27 – 2018-7-07.

### Notes

The sampled material was collected in the period from 27 May 2014 to 07 July 2018

## Collection data

### Collection name

InBIO Barcoding Initiative

### Collection identifier

4ec2b246-f5fa-4b90-9a8d-ddafc2a3f970

### Specimen preservation method

“Alcohol”

### Curatorial unit

Voucher tube - 1 to 203, DNA extractions - 1 to 203

## Usage rights

### Use license

Creative Commons Public Domain Waiver (CC-Zero)

## Data resources

### Data package title

The InBIO Barcoding Initiative Database: Diptera 01

### Resource link

dx.doi.org/10.5883/DS-IBIDP01

### Number of data sets

1

### Data set 1.

#### Data set name

DS-IBIDP01 IBI Diptera 01

#### Data format

dwc, xml, tsv, fasta

#### Number of columns

35

#### Download URL

http://www.boldsystems.org/index.php/Public_SearchTerms?query=DS-IBIDP01

#### Description

The InBIO Barcoding Initiative Database: Diptera 01 dataset can be downloaded from the Public Data Portal of BOLD (dx.doi.org/10.5883/DS-IBIDP01) in different formats (data as dwc, xml or tsv, and sequences as fasta files). Alternatively, BOLD users can log-in and access the dataset via the Workbench platform of BOLD. All records are also searchable within BOLD, using the search function of the database.

The InBIO Barcoding Initiative will continue sequencing Diptera for the BOLD database, with the ultimate goal of comprehensive coverage. The version of the dataset, at the time of writing the manuscript, is included as Suppl. materials 2, 3, 4 in the form of two text files for record information as downloaded from BOLD, one text file with the collecting and identification data in Darwin Core Standard format (downloaded from GBIF) and of a fasta file containing all sequences as downloaded from BOLD.

It should be noted that, as the BOLD database is not compliant with the Darwin Core Standard format, the Darwin Core formatted file (dwc) that can be downloaded from BOLD is not strictly Darwin Core formatted. For a proper Darwin Core formatted file, see http://ipt.gbif.pt/ipt/resource?r=ibi_diptera_i&amp;v=1.0 (Suppl. material 3).

Column labels below follow the labels downloaded in the tsv format. Columns with no content in our dataset are left out in the list below.

**Data set 1.** 

Column label	Column description
processid	Unique identifier for the sample
sampleid	Identifier for the sample being sequenced, i.e. IBI catalogue number at Cibio-InBIO, Porto University. Often identical to the "Field ID" or "Museum ID"
recordID	Identifier for specimen assigned in the field
catalognum	Catalogue number
fieldnum	Field number
institution_storing	The full name of the institution that has physical possession of the voucher specimen
bin_uri	Barcode Index Number system identifier
phylum_taxID	Phylum taxonomic numeric code
phylum_name	Phylum name
class_taxID	Class taxonomic numeric code
class_name	Class name
order_taxID	Order taxonomic numeric code
order_name	Order name
family_taxID	Family taxonomic numeric code
family_name	Family name
subfamily_taxID	Subfamily taxonomic numeric code
subfamily_name	Subfamily name
genus_taxID	Genus taxonomic numeric code
genus_name	Genus name
species_taxID	Species taxonomic numeric code
species_name	Species name
identification_provided_by	Full name of primary individual who assigned the specimen to a taxonomic group
identification_method	The method used to identify the specimen
voucher_status	Status of the specimen in an accessioning process (BOLD controlled vocabulary)
tissue_type	A brief description of the type of tissue or material analysed
collectors	The full or abbreviated names of the individuals or team responsible for collecting the sample in the field
lifestage	The age class or life stage of the specimen at the time of sampling
sex	The sex of the specimen
lat	The geographical latitude (in decimal degrees) of the geographic centre of a location
lon	The geographical longitude (in decimal degrees) of the geographic centre of a location
elev	Elevation of sampling site (in metres above sea level)
country	The full, unabbreviated name of the country where the organism was collected
province_state	The full, unabbreviated name of the province ("Distrito" in Portugal) where the organism was collected
region	The full, unabbreviated name of the municipality ("Concelho" in Portugal) where the organism was collected
exactsite	Additional name/text description regarding the exact location of the collection site relative to a geographic relevant landmark

## Supplementary Material

1A6AE9E5-D722-54DA-B720-9C7CB62A95D4 10.3897/BDJ.8.e49985.suppl1 Supplementary material 1 References used for morphological identification Data type: References
Brief description: References used for morphological identification
File: oo_369593.txt https://binary.pensoft.net/file/369593 Rui Andrade

E3F7460F-4A3A-5841-AAFD-FE1B279EE58F 10.3897/BDJ.8.e49985.suppl2 Supplementary material 2 IBI- Diptera 01 library - Specimen details Data type: Record information - specimen data
Brief description: The file includes information about all records in BOLD for the IBI- Diptera 01 library. It contains collecting and identification data. The data are as downloaded from BOLD, without further processing.
File: oo_369598.txt https://binary.pensoft.net/file/369598 Sónia Ferreira, Rui Andrade, Ana Rita Gonçalves, Pedro Sousa, Pedro Beja

8CC4D380-11EF-5C21-AEF2-728C217596B3 10.3897/BDJ.8.e49985.suppl3 Supplementary material 3 IBI- Diptera 01 library - Specimen details - Darwin Core Standard Data type: Record information - specimen data in Darwin Core Standard format
Brief description: The file includes information about all records in BOLD for the IBI- Diptera 01 library. It contains collecting and identification data. The data are downloaded from GBIF, without further processing.
File: oo_379613.txt https://binary.pensoft.net/file/379613 Sónia Ferreira, Rui Andrade, Ana Rita Gonçalves, Pedro Sousa, Pedro Beja

2E4F7974-7BFE-5F35-9F68-1F5CE59DE12B 10.3897/BDJ.8.e49985.suppl4 Supplementary material 4 IBI- Diptera 01 library - DNA sequences Data type: Genomic data, DNA sequences
Brief description: COI sequences in fasta format. Each sequence is identified by the BOLD ProcessID, species name, marker and GenBank accession number, separated by pipe. The data are as downloaded from BOLD.
File: oo_379616.fas https://binary.pensoft.net/file/379616 Sónia Ferreira, Rui Andrade, Ana Rita Gonçalves, Pedro Sousa, Pedro Beja

## Figures and Tables

**Figure 1a.**
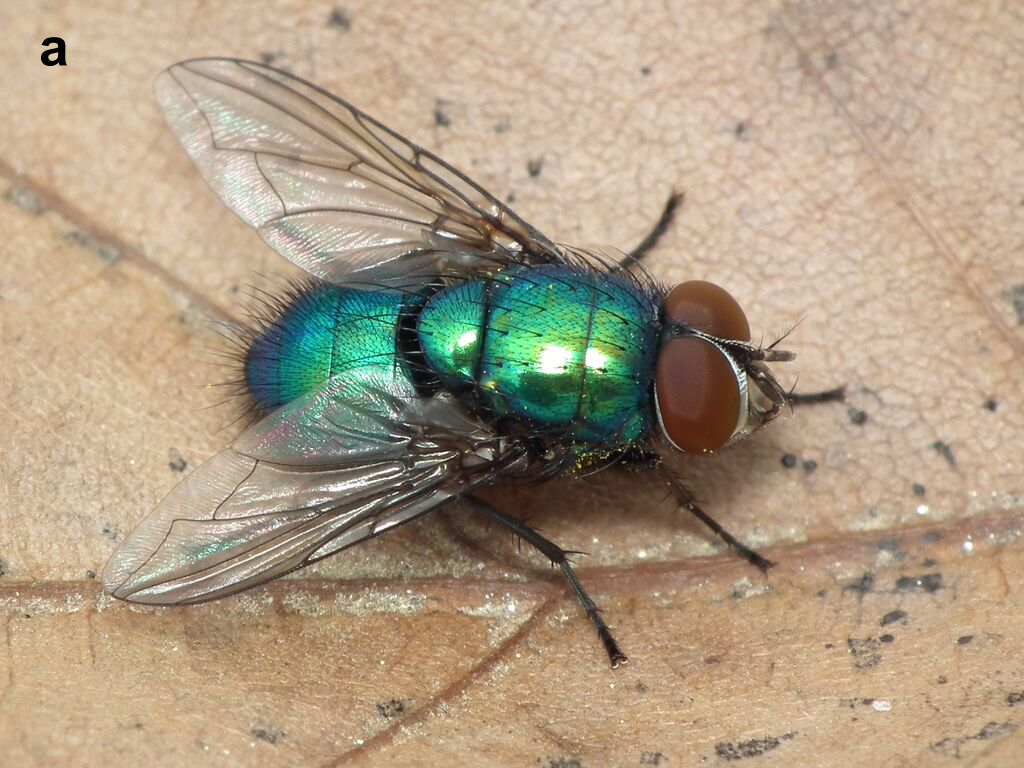
*Lucilia caesar*, Lordelo do Ouro - Porto, BOLD:IBIDP083-19, GenBank: MN868752

**Figure 1b.**
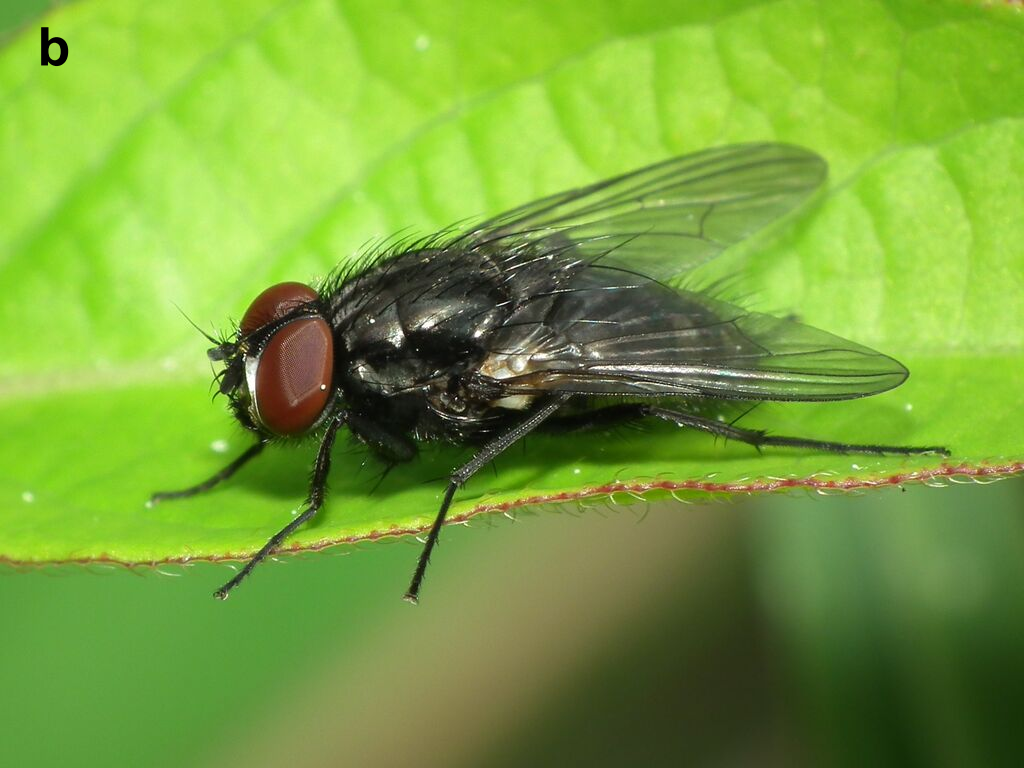
*Hydrotaea dentipes*, Campo - Valongo, BOLD: IBIDP136-19, GenBank: MN868754

**Figure 1c.**
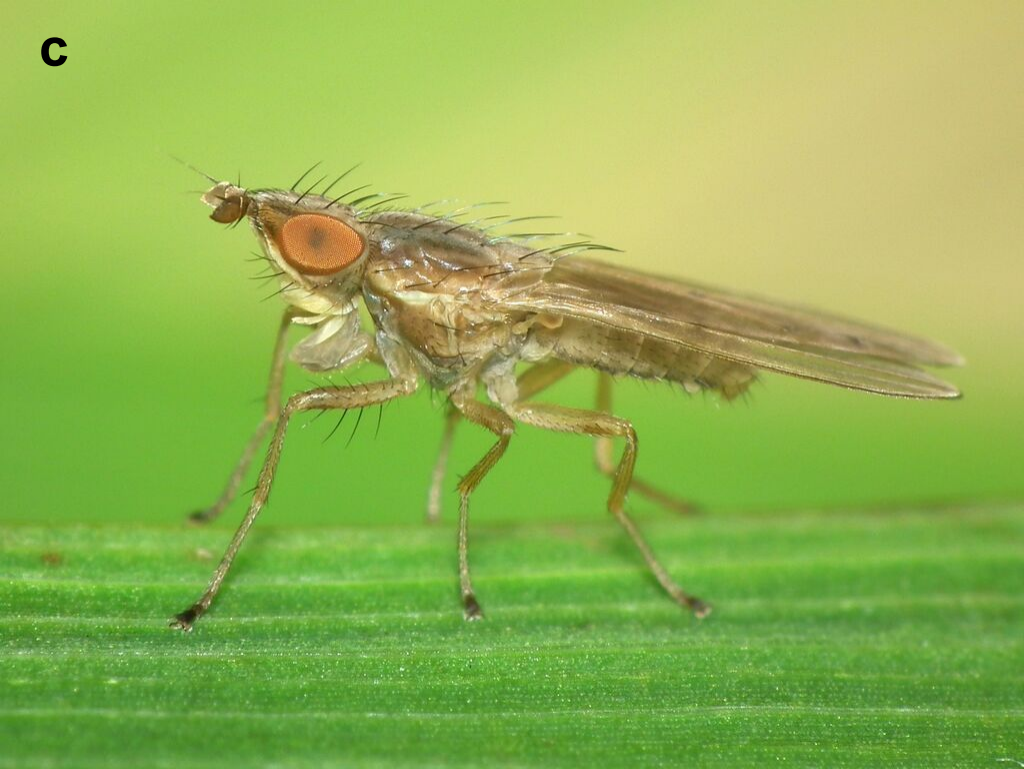
*Trigonometopus frontalis*, Canelas - Estarreja, BOLD: IBIDP099-19, GenBank: MN868863

**Figure 1d.**
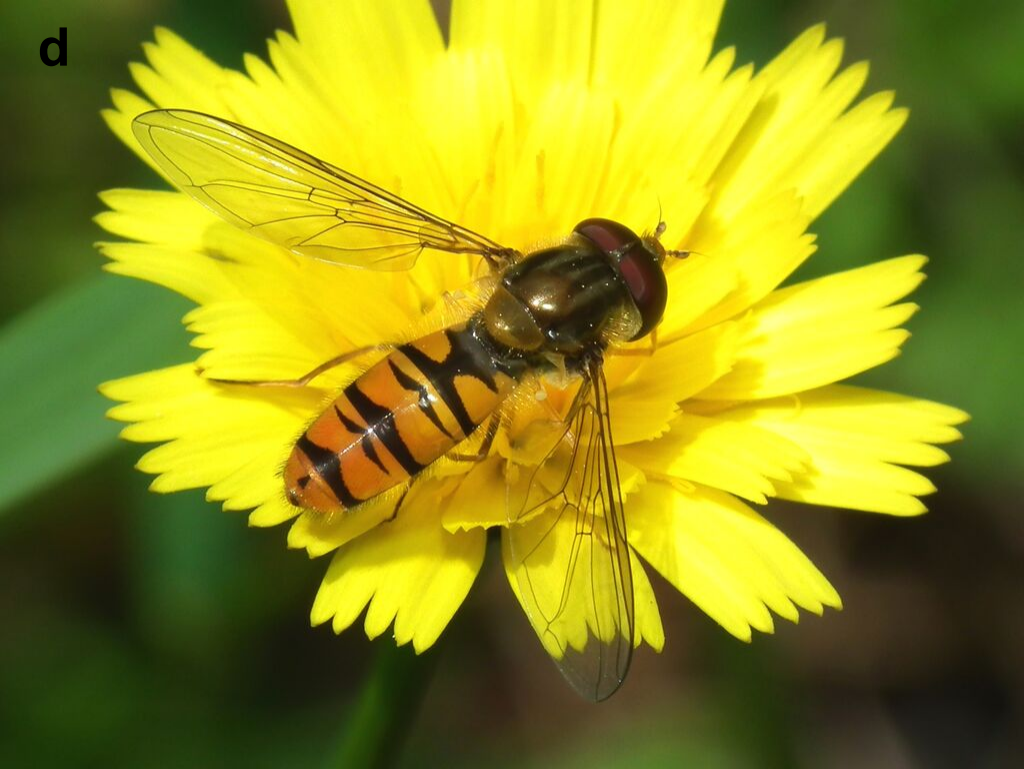
*Episyrphus balteatus*, Vale de Algoso - Vimioso, BOLD: IBIDP015-19, GenBank: MN868781

**Figure 1e.**
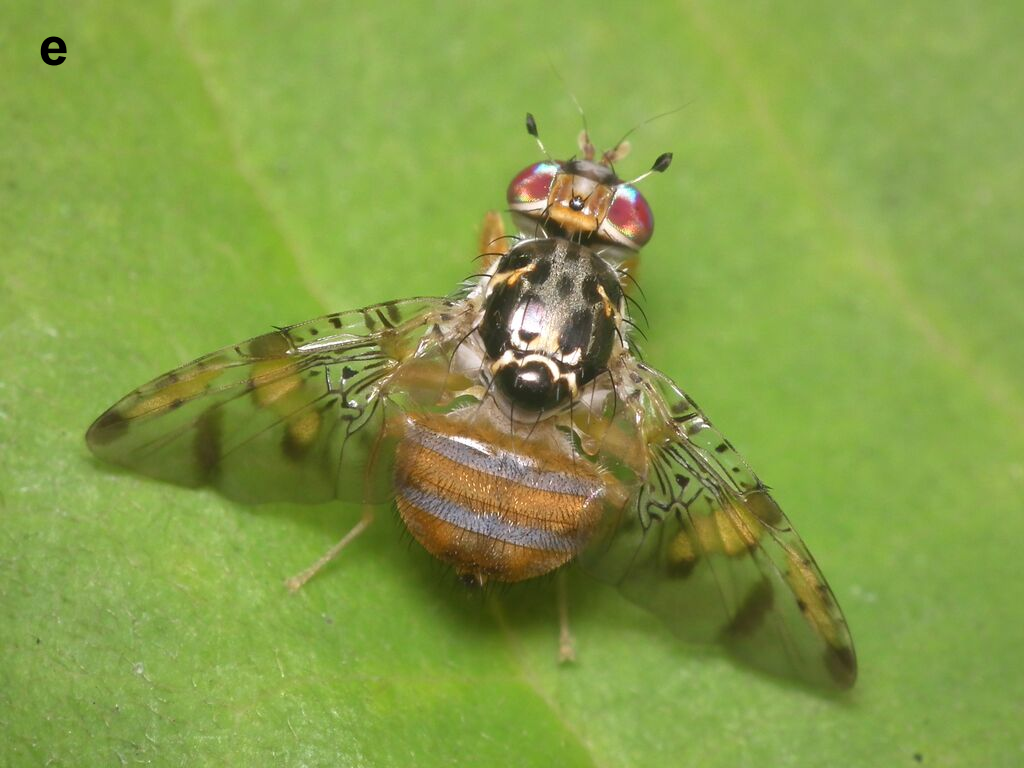
*Ceratitis capitata*, Gilmonde - Barcelos, BOLD: IBIDP140-19, GenBank: MN868854

**Table 1.** Number of specimens collected per Portuguese district and corresponding percentage. The number of recorded specimens also corresponds to the number of species recorded per district, as each species was collected only once in each district.

**District**	**Specimens (n)**	**Specimens (%)**
Bragança	79	38.9%
Aveiro	51	25.1%
Porto	38	18.7%
Braga	21	10.3%
Vila Real	6	3.0%
Castelo Branco	4	2.0%
Viana do Castelo	2	1.0%
Guarda	1	0.5%
not known	1	0.5%
**Total**	203	100%

**Table 2.** List of species that were collected and DNA barcoded within this project. * Indicate species without DNA barcode prior to this study, '' indicates species with less than 10 sequences prior to this study.

**Family**	**Species**	**IBI code**	**BOLD code**	**BOLD BIN**	**GenBank**
Anisopodidae	*Sylvicola cinctus* (Fabricius, 1787)	INV04902	IBIDP034-19	BOLD:AAG1996	MN868840
Anthomyiidae	*Anthomyia imbrida* Rondani, 1866''	INV00551	IBIDP006-19	BOLD:ABX9294	MN868736
Anthomyiidae	*Anthomyia pluvialis* (Linnaeus, 1758)	INV00817	IBIDP144-19	BOLD:ADH9310	MN868903
Anthomyiidae	*Subhylemyia longula* (Fallen, 1824)''	INV00555	IBIDP007-19	BOLD:AAP8836	MN868762
Asilidae	*Molobratia teutonus* (Linnaeus, 1767)''	INV07407	IBIDP391-19	BOLD:ADF9805	MN868906
Bibionidae	*Dilophus febrilis* (Linnaeus, 1758)	INV04935	IBIDP055-19	BOLD:ACI4790	MN868809
Bombyliidae	*Anthrax anthrax* (Schrank, 1781)''	INV04944	IBIDP063-19	BOLD:ADR8193	MN868791
Bombyliidae	*Exhyalanthrax afer* (Fabricius, 1794)*	INV04951	IBIDP151-19	BOLD:ADU1899	MN868734
Bombyliidae	Hemipenthes morio (Linnaeus, 1758)''	INV04959	IBIDP070-19	BOLD:ACS6268	MN868882
Calliphoridae	*Calliphora vicina* Robineau-Desvoidy, 1830	INV00609, INV01920, INV05001, INV05029	IBIDP009-19, IBIDP018-19, IBIDP087-19, IBIDP167-19	BOLD:AAB6579	MN868831, MN868732, MN868823, MN868797
Calliphoridae	*Calliphora vomitoria* (Linnaeus, 1758)	INV00176, INV02884, INV05036, INV07378	IBIDP001-19, IBIDP024-19, IBIDP107-19, IBIDP381-19	BOLD:AAA8931	MN868884, MN868856, MN868898, MN868853
Calliphoridae	*Chrysomya albiceps* (Wiedemann, 1819)	INV05008, INV05075	IBIDP092-19, IBIDP125-19	BOLD:ABX6432	MN868731, MN868796
Calliphoridae	Lucilia caesar (Linnaeus, 1758)	INV04986	IBIDP083-19	BOLD:AAA7470	MN868868
Calliphoridae	Lucilia sericata (Meigen, 1826)	INV04987, INV05048	IBIDP084-19, IBIDP112-19	BOLD:AAA6618	MN868905, MN868854
Canacidae	*Canace nasica* (Haliday, 1839)*	INV04995	IBIDP161-19	BOLD:ADT0044	MN868786
Canacidae	Tethina illota (Haliday, 1838)''	INV05007	IBIDP091-19	BOLD:ABA8670	MN868899
Chloropidae	*Camarota curvipennis* (Latreille, 1805)*	INV04955	IBIDP152-19	BOLD:ACP6247	MN868844
Chloropidae	*Cryptonevra flavitarsis* (Meigen, 1830)''	INV04994	IBIDP086-19	BOLD:ACB6675	MN868783
Chloropidae	*Diplotoxa messoria* (Fallen, 1820)''	INV05017, INV05081	IBIDP098-19, IBIDP127-19	BOLD:ACD3417	MN868807, MN868793
Chloropidae	*Elachiptera bimaculata* (Loew, 1845)*	INV04990	IBIDP158-19	BOLD:ADU0665	MN868888
Chloropidae	*Elachiptera megaspis* (Loew, 1858)	INV05080, INV07368	IBIDP174-19,IBIDP374-19	BOLD:ADU0581	MN868842, MN868711
Chloropidae	*Eutropha albipilosa* (Becker, 1908)*	INV04991	IBIDP159-19	BOLD:ADS5005	MN868764
Chloropidae	*Eutropha fulvifrons* (Haliday, 1833)*	INV04992	IBIDP160-19	BOLD:ADT0252	MN868855
Chloropidae	*Lasiochaeta pubescens* Thalhammer, 1898*	INV05082	IBIDP175-19	BOLD:ACP6247	MN868876
Chloropidae	*Polyodaspis sulcicollis* (Meigen, 1838)*	INV04989	IBIDP157-19	BOLD:ADS6761	MN868863
Chloropidae	*Thaumatomyia elongatula* (Becker, 1910)*	INV05083	IBIDP176-19	BOLD:ADV5221	MN868862
Chloropidae	*Trachysiphonella ruficeps* (Macquart, 1835)''	INV04996	IBIDP162-19	BOLD:ACP4572	MN868766
Conopidae	*Conops flavipes* Linnaeus, 1758''	INV05010	IBIDP164-19	BOLD:AAJ7146	MN868871
Diastatidae	*Diastata adusta* Meigen, 1830*	INV05066	IBIDP172-19	BOLD:ADS5309	MN868880
Dolichopodidae	*Campsicnemus scambus* (Fallen, 1823)''	INV04938	IBIDP058-19	BOLD:ACP5225	MN868902
Dolichopodidae	*Campsicnemus umbripennis* Loew, 1856	INV07394	IBIDP387-19	BOLD:ACQ9843	MN868739
Dolichopodidae	*Gymnopternus cupreus* (Fallen, 1823)*	INV04909	IBIDP147-19	BOLD:AAZ8613	MN868838
Dolichopodidae	*Machaerium maritimae* Haliday, 1832*	INV05105	IBIDP179-19	BOLD:ADT1791	MN868724
Dolichopodidae	*Sciapus platypterus* (Fabricius, 1805)	INV04907	IBIDP037-19	BOLD:ABU9505	MN868817
Drosophilidae	*Drosophila suzukii* (Matsumura, 1931)	INV05107	IBIDP135-19	BOLD:AAC2499	MN868726
Drosophilidae	*Leucophenga maculata* (Dufour, 1839)	INV05006	IBIDP090-19	BOLD:ACC9004	MN868775
Drosophilidae	*Phortica variegata* (Fallen, 1823)''	INV04906	IBIDP036-19	BOLD:ADP2209	MN868851
Ephydridae	*Discomyza incurva* Fallen, 1823	INV04956	IBIDP069-19	BOLD:ABA8754	MN868839
Ephydridae	*Hecamede albicans* (Meigen, 1830)''	INV04993	IBIDP085-19	BOLD:ABA8686	MN868716
Ephydridae	*Psilopa nitidula* (Fallen, 1813)''	INV04984	IBIDP081-19	BOLD:AAG6948	MN868834
Fanniidae	*Fannia canicularis* (Linnaeus, 1761)	INV00177, INV04943	IBIDP002-19, IBIDP062-19	BOLD:AAF7101	MN868723, MN868818
Fanniidae	*Fannia lustrator* (Harris, 1780)	INV07364	IBIDP372-19	BOLD:ACB3656	MN868758
Heleomyzidae	*Suillia variegata* (Loew, 1862)	INV00970, INV04948	IBIDP011-19, IBIDP065-19	BOLD:ABY1201	MN868749, MN868727
Hippoboscidae	*Hippobosca equina* Linnaeus, 1758''	INV07388	IBIDP385-19	BOLD:AAX0882	MN868873
Hybotidae	*Hybos culiciformis* (Fabricius, 1775)	INV01283, INV01808, INV05078	IBIDP013-19, IBIDP014-19, IBIDP173-19	BOLD:ACD8518	MN868781, MN868800, MN868846
Lauxaniidae	*Calliopum simillimum* (Collin, 1933)	INV07387	IBIDP384-19	BOLD:ACP8933	MN868801
Lauxaniidae	*Minettia longipennis* (Fabricius, 1794)	INV04908	IBIDP038-19	BOLD:ACB3141	MN868788
Lauxaniidae	*Prosopomyia pallida* Loew, 1856*	INV04946	IBIDP150-19	BOLD:ADV2313	MN868794
Lauxaniidae	Trigonometopus frontalis (Meigen, 1830)''	INV05019	IBIDP099-19	BOLD:ACS3478	MN868881
Lonchaeidae	*Lamprolonchaea smaragdi* (Walker, 1849)''	INV05120	IBIDP184-19	BOLD:ACO9728	MN868843
Lonchaeidae	*Silba fumosa* (Egger, 1862)*	INV04985	IBIDP082-19	BOLD:ACQ9578	MN868841
Micropezidae	*Micropeza lateralis* Meigen, 1826''	INV01282	IBIDP145-19	BOLD:ADC0694	MN868910
Muscidae	*Coenosia atra* Meigen, 1830	INV04968, INV05090	IBIDP075-19, IBIDP131-19	BOLD:ACP3222	MN868737, MN868850
Muscidae	*Coenosia attenuata* Stein, 1903	INV05122	IBIDP142-19	BOLD:AAD7633	MN868861
Muscidae	*Coenosia tigrina* (Fabricius, 1775)	INV04928	IBIDP052-19	BOLD:AAB5609	MN868826
Muscidae	*Dasyphora albofasciata* (Macquart & Berthelot, 1839)*	INV05100	IBIDP178-19	BOLD:ADS1325	MN868827
Muscidae	*Graphomya maculata* (Scopoli, 1763)	INV04919	IBIDP046-19	BOLD:ABV4895	MN868806
Muscidae	Helina evecta (Harris, 1780)	INV05054	IBIDP115-19	BOLD:ACB3279	MN868729
Muscidae	Hydrotaea dentipes (Fabricius, 1805)	INV05110	IBIDP136-19	BOLD:AAI8769	MN868883
Muscidae	*Lispe kowarzi* Becker, 1903''	INV04975	IBIDP155-19	BOLD:ACA1246	MN868872
Muscidae	*Lispocephala mikii* (Strobl, 1893)''	INV04918	IBIDP148-19	BOLD:ADT6256	MN868912
Muscidae	*Musca autumnalis* De Geer, 1776	INV04936	IBIDP056-19	BOLD:AAA3187	MN868877
Muscidae	*Musca domestica* Linnaeus, 1758	INV05087	IBIDP130-19	BOLD:AAA6020	MN868773
Muscidae	*Musca tempestiva* Fallen, 1817*	INV04974	IBIDP154-19	BOLD:ACO3332	MN868751
Muscidae	*Muscina levida* (Harris, 1780)	INV04940	IBIDP059-19	BOLD:AAB8817	MN868769
Muscidae	*Muscina pascuorum* (Meigen, 1826)	INV04941	IBIDP060-19	BOLD:AAG1714	MN868746
Muscidae	*Muscina stabulans* (Fallen, 1817)	INV05015, INV05106	IBIDP096-19, IBIDP134-19	BOLD:AAF6582	MN868720, MN868760
Muscidae	*Neomyia cornicina* (Fabricius, 1781)	INV04922	IBIDP048-19	BOLD:AAH3032	MN868811
Muscidae	*Orchisia costata* (Meigen, 1826)	INV05022	IBIDP101-19	BOLD:ABX0213	MN868830
Muscidae	*Phaonia fuscata* (Fallen, 1825)	INV04952	IBIDP067-19	BOLD:ACB5198	MN868753
Muscidae	*Phaonia pallida* (Fabricius, 1787)	INV05045	IBIDP110-19	BOLD:ABW3852	MN868712
Muscidae	*Polietes meridionalis* (Peris & Llorente, 1963)	INV04964, INV05055	IBIDP072-19, IBIDP116-19	BOLD:AAY2766	MN868770, MN868865
Muscidae	*Pyrellia vivida* Robineau-Desvoidy, 1830*	INV04970	IBIDP153-19	BOLD:ADT8849	MN868828
Muscidae	*Schoenomyza litorella* (Fallen, 1823)	INV02961, INV04969	IBIDP029-19, IBIDP076-19	BOLD:ABW5574	MN868787, MN868835
Nemestrinidae	*Fallenia fasciata* (Fabricius, 1805)''	INV07404	IBIDP390-19	BOLD:ADR9796	MN868810
Opetiidae	Opetia nigra Meigen, 1830''	INV04942	IBIDP061-19	BOLD:ACD2824	MN868780
Opomyzidae	*Opomyza petrei* Mesnil, 1934	INV04953, INV05077	IBIDP068-19, IBIDP126-19	BOLD:AAG1210	MN868887, MN868745
Pallopteridae	*Toxoneura muliebris* (Harris, 1780)*	INV05115	IBIDP139-19	BOLD:ACQ9316	MN868763
Periscelididae	Periscelis fugax*	INV05109	IBIDP181-19	BOLD:ADU2556	MN868715
Platystomatidae	*Rivellia syngenesiae* (Fabricius, 1781)	INV04931, INV07385	IBIDP054-19, IBIDP383-19	BOLD:AAX8866	MN868768, MN868792
Psychodidae	*Clogmia albipunctata* (Williston, 1893)	INV05093	IBIDP177-19	BOLD:ACS5395	MN868879
Ptychopteridae	*Ptychoptera albimana* (Fabricius, 1787)	INV07376	IBIDP379-19	BOLD:ACL4518	MN868771
Rhiniidae	*Rhyncomya columbina* (Meigen, 1824)*	INV04981	IBIDP156-19	BOLD:ADU6456	MN868859
Rhiniidae	*Rhyncomya felina* (Fabricius, 1794)*	INV05057	IBIDP171-19	BOLD:ADV4310	MN868896
Rhiniidae	Stomorhina lunata (Fabricius, 1805)''	INV04929, INV05044	IBIDP053-19, IBIDP109-19	BOLD:ACD9536	MN868816, MN868892
Rhinophoridae	*Melanophora roralis* (Linnaeus, 1758)	INV04949	IBIDP066-19	BOLD:AAG6862	MN868885
Rhinophoridae	*Oplisa aterrima* (Strobl, 1899)''	INV04926	IBIDP149-19	BOLD:ADU0356	MN868890
Sarcophagidae	*Sarcophaga crassipalpis* Macquart, 1839	INV00698	IBIDP010-19	BOLD:AAC1709	MN868822
Sarcophagidae	*Sarcophaga longestylata* Strobl, 1906*	INV05116	IBIDP182-19	BOLD:ADV1383	MN868891
Scathophagidae	*Norellia tipularia* (Fabricius, 1794)''	INV07396	IBIDP388-19	BOLD:ACE2160	MN868870
Scathophagidae	*Norellisoma spinimanum* (Fallen, 1819)	INV04924	IBIDP049-19	BOLD:ACD3336	MN868721
Scathophagidae	*Scathophaga stercoraria* (Linnaeus, 1758)	INV00527, INV02883, INV04913, INV07377	IBIDP004-19, IBIDP023-19, IBIDP041-19, IBIDP380-19	BOLD:AAD0853	MN868858, MN868799, MN868719, MN868829
Sciomyzidae	*Euthycera alaris* Vala, 1983*	INV01287	IBIDP146-19	BOLD:ADV6563	MN868813
Sciomyzidae	*Euthycera cribrata* (Rondani, 1868)*	INV05031	IBIDP168-19	BOLD:ADU4073	MN868789
Sciomyzidae	*Ilione trifaria* (Loew, 1847)''	INV05084, INV05024	IBIDP128-19, IBIDP102-19	BOLD:ADR6909	MN868748, MN868717
Sciomyzidae	*Pherbellia cinerella* (Fallen, 1820)	INV05004	IBIDP089-19	BOLD:ACB5604	MN868757
Sciomyzidae	*Pherbellia dorsata* (Zetterstedt, 1846)''	INV05061	IBIDP119-19	BOLD:ACD6777	MN868901
Sciomyzidae	*Pherbina mediterranea* Mayer, 1953*	INV05117	IBIDP183-19	BOLD:ADU0215	MN868714
Stratiomyidae	*Chloromyia formosa* (Scopoli, 1763)	INV04925, INV07375	IBIDP050-19, IBIDP378-19	BOLD:ABU9940	MN868798, MN868735
Stratiomyidae	*Hermetia illucens* (Linnaeus, 1758)	INV01259, INV04643, INV05003, INV05035	IBIDP012-19, IBIDP032-19, IBIDP088-19, IBIDP106-19	BOLD:AAG3698, BOLD:AAD0622	MN868718, MN868889, MN868825, MN868864
Syrphidae	*Chalcosyrphus nemorum* (Fabricius, 1805)	INV05104	IBIDP133-19	BOLD:AAG6762	MN868908
Syrphidae	*Chrysotoxum intermedium* Meigen, 1822	INV05041	IBIDP108-19	BOLD:AAE9233	MN868747
Syrphidae	*Dasysyrphus albostriatus* (Fallen, 1817)	INV07391	IBIDP386-19	BOLD:AAL1242	MN868722
Syrphidae	Episyrphus balteatus (De Geer, 1776)	INV01909, INV07402	IBIDP015-19, IBIDP389-19	BOLD:AAC6833	MN868761, MN868738
Syrphidae	*Eristalinus aeneus* (Scopoli, 1763)	INV05013	IBIDP095-19	BOLD:AAF3600	MN868802
Syrphidae	*Eristalinus sepulchralis* (Linnaeus, 1758)	INV05025, INV05072	IBIDP103-19, IBIDP123-19	BOLD:AAZ5387	MN868777, MN868819
Syrphidae	*Eristalinus taeniops* (Wiedemann, 1818)''	INV01912, INV04912	IBIDP017-19, IBIDP040-19	BOLD:ACL5309	MN868857, MN868779
Syrphidae	*Eristalis arbustorum* (Linnaeus, 1758)	INV01948, INV02915, INV04917, INV05071	IBIDP021-19, IBIDP027-19, IBIDP045-19, IBIDP122-19	BOLD:AAA8223	MN868782, MN868832, MN868878, MN868725
Syrphidae	*Eristalis pertinax* (Scopoli, 1763)	INV04965	IBIDP073-19	BOLD:AAQ3585	MN868733
Syrphidae	*Eristalis similis* (Fallen, 1817)	INV01910, INV04915	IBIDP016-19, IBIDP043-19	BOLD:AAY9892	MN868866, MN868894
Syrphidae	*Eristalis tenax* (Linnaeus, 1758)	INV01937, INV02914, INV04916	IBIDP019-19, IBIDP026-19, IBIDP044-19	BOLD:AAB0391	MN868900, MN868756, MN868909
Syrphidae	*Ferdinandea cuprea* (Scopoli, 1763)	INV05021	IBIDP100-19	BOLD:AAJ0402	MN868897
Syrphidae	*Helophilus pendulus* (Linnaeus, 1758)	INV04914	IBIDP042-19	BOLD:AAI6747	MN868765
Syrphidae	*Melanostoma mellinum* (Linnaeus, 1758)	INV04977	IBIDP078-19	BOLD:AAB2866	MN868833
Syrphidae	*Milesia crabroniformis* (Fabricius, 1775)''	INV04153, INV05016	IBIDP031-19, IBIDP097-19	BOLD:ADS0144	MN868804, MN868904
Syrphidae	*Myathropa florea* (Linnaeus, 1758)	INV01938, INV04963	IBIDP020-19, IBIDP071-19	BOLD:AAP9713, BOLD:ADQ8445	MN868821, MN868847
Syrphidae	*Paragus quadrifasciatus* Meigen, 1822	INV00560	IBIDP008-19	BOLD:ACG5063	MN868744
Syrphidae	*Platycheirus rosarum* (Fabricius, 1787)	INV04911, INV05074	IBIDP039-19, IBIDP124-19	BOLD:AAG4683	MN868812, MN868740
Syrphidae	*Scaeva pyrastri* (Linnaeus, 1758)	INV05070	IBIDP121-19	BOLD:AAF2374	MN868785
Syrphidae	*Sphaerophoria rueppelli* (Wiedemann, 1830)*	INV05058	IBIDP117-19	BOLD:AAA7374	MN868820
Syrphidae	Sphaerophoria scripta (Linnaeus, 1758)	INV02901, INV04900	IBIDP025-19, IBIDP033-19	BOLD:AAA7374	MN868774, MN868784
Syrphidae	*Syritta flaviventris* Macquart, 1842	INV04973	IBIDP077-19	BOLD:AAG4663	MN868803
Syrphidae	*Syritta pipiens* (Linnaeus, 1758)	INV04937, INV05042, INV05097	IBIDP057-19, IBIDP170-19, IBIDP132-19	BOLD:AAC6291	MN868778, MN868754, MN868869
Syrphidae	*Syrphus vitripennis* Meigen, 1822	INV07372	IBIDP377-19	BOLD:AAB5577	MN868776
Syrphidae	*Volucella zonaria* (Poda, 1761)	INV04979	IBIDP080-19	BOLD:AAH7785	MN868837
Tabanidae	*Chrysops caecutiens* (Linnaeus, 1758)	INV07371	IBIDP376-19	BOLD:ADZ0489	MN868849
Tabanidae	*Chrysops viduatus* (Fabricius, 1794)''	INV04978	IBIDP079-19	BOLD:ACB5910	MN868728
Tabanidae	*Haematopota ocelligera* (Krober, 1922)*	INV05023	IBIDP166-19	BOLD:ADU0467	MN868772
Tachinidae	*Besseria dimidiata* (Zetterstedt, 1844)*	INV04998	IBIDP163-19	BOLD:ADT0060	MN868755
Tachinidae	*Compsilura concinnata* (Meigen, 1824)	INV05119	IBIDP141-19	BOLD:ADK6725	MN868913
Tachinidae	*Cylindromyia bicolor* (Olivier, 1811)	INV02245, INV04921	IBIDP022-19, IBIDP047-19	BOLD:AAN3295	MN868867, MN868808
Tachinidae	*Cylindromyia rufipes* (Meigen, 1824)''	INV05014	IBIDP165-19	BOLD:AAU6684	MN868795
Tachinidae	*Dexia rustica* (Fabricius, 1775)''	INV04945	IBIDP064-19	BOLD:ACD9618	MN868750
Tachinidae	*Ectophasia crassipennis* (Fabricius, 1794)''	INV05026	IBIDP104-19	BOLD:ADJ6473	MN868852
Tachinidae	*Eloceria delecta* (Meigen, 1824)''	INV04905	IBIDP035-19	BOLD:ACA9834	MN868895
Tachinidae	*Frontina laeta* (Meigen, 1824)''	INV05027	IBIDP105-19	BOLD:ABW4362	MN868874
Tachinidae	*Leskia aurea* (Fallen, 1820)''	INV05011	IBIDP094-19	BOLD:AAV7830	MN868759
Tachinidae	*Microphthalma europaea* Egger, 1860	INV05111	IBIDP137-19	BOLD:AAU6726	MN868814
Tachinidae	*Mintho rufiventris* (Fallen, 1817)	INV05053	IBIDP114-19	BOLD:AAX3938	MN868730
Tachinidae	*Nemoraea pellucida* (Meigen, 1824)''	INV05108	IBIDP180-19	BOLD:ACD9580	MN868848
Tachinidae	*Peleteria rubescens* (Robineau-Desvoidy, 1830)''	INV05113	IBIDP138-19	BOLD:AAZ5252	MN868713
Tachinidae	*Peribaea tibialis* (Robineau-Desvoidy, 1851)	INV07382	IBIDP382-19	BOLD:ACB1892	MN868907
Tachinidae	*Periscepsia carbonaria* (Panzer, 1798)	INV05046, INV05085	IBIDP111-19, IBIDP129-19	BOLD:ACD9481	MN868815, MN868767
Tachinidae	*Phasia pusilla* Meigen, 1824	INV07360	IBIDP371-19	BOLD:AAY2270	MN868790
Tachinidae	*Tachina lurida* (Fabricius, 1781)''	INV07369	IBIDP375-19	BOLD:ACA9827	MN868845
Tachinidae	*Tachina magnicornis* (Zetterstedt, 1844)	INV04967, INV05052	IBIDP074-19, IBIDP113-19	BOLD:AAN9512	MN868752, MN868741
Tachinidae	*Triarthria setipennis* (Fallen, 1810)	INV00529	IBIDP005-19	BOLD:ADK0727	MN868911
Tachinidae	*Zophomyia temula* (Scopoli, 1763)''	INV04927	IBIDP051-19	BOLD:ACD1492	MN868886
Tephritidae	Ceratitis capitata (Wiedemann, 1824)	INV05118	IBIDP140-19	BOLD:AAA3297	MN868742
Trichoceridae	*Trichocera saltator* (Harris, 1776)	INV06188	IBIDP143-19	BOLD:ACQ9998	MN868805
Ulidiidae	*Ceroxys urticae* (Linnaeus, 1758)''	INV05059	IBIDP118-19	BOLD:AAI7007	MN868893
Ulidiidae	*Myennis octopunctata* (Coquebert, 1798)''	INV00518	IBIDP003-19	BOLD:AAG7348	MN868824
Ulidiidae	*Physiphora alceae* (Preyssler, 1791)	INV03580	IBIDP030-19	BOLD:ABA5170	MN868875
Ulidiidae	*Ulidia apicalis* Meigen, 1826''	INV02952, INV05037, INV07366	IBIDP028-19, IBIDP169-19, IBIDP373-19	BOLD:ABX4821	MN868743, MN868836, MN868860

## References

[B5469595] Andrade R., Gonçalves A. R (2014). Acartophthalmidae, Pseudopomyzidae and Xylomyidae – Three families of Diptera new to the Portuguese fauna.. Boletín Sociedad Entomológica Aragonesa.

[B5526361] Baird Donald J., Pascoe Timothy J., Zhou Xin, Hajibabaei Mehrdad (2011). Building freshwater macroinvertebrate DNA-barcode libraries from reference collection material: formalin preservation vs specimen age. Journal of the North American Benthological Society.

[B5469680] Carles-Tolrá M. (2002). Catálogo de los Diptera de España, Portugal y Andorra (Insecta)..

[B5465249] Corley Martin, Ferreira Sónia (2017). DNA Barcoding reveals sexual dimorphism in *Isotrias
penedana* Trematerra, 2013 (Lepidoptera: Tortricidae, Chlidanotinae). Zootaxa.

[B5465269] Corley Martin, Ferreira Sónia, Mata V. A. (2019). *Ypsolopha
rhinolophi* sp. nov. (Lepidoptera: Ypsolophidae), a new species from Portugal and France unveiled by bats. Zootaxa.

[B5469747] Corley M. F.V., Buchner P., Ferreira S. (2019). *Depressaria
infernella* Corley & Buchner, a new Iberian species of the *Depressaria
douglasella* group (Lepidoptera: Depressariidae.. SHILAP Revista de Lepidopterología.

[B5465259] Corley Martin F. V., Ferreira Sónia, Lvovsky Alexander L., Rosete Jorge (2017). *Borkhausenia
crimnodes* Meyrick, 1912 (Lepidoptera, Oecophoridae), a southern hemisphere species resident in Portugal. Nota Lepidopterologica.

[B5469784] Ebejer M. J., Andrade R. (2015). The Chloropidae (Diptera: Brachycera) of mainland Portugal with description of a new species of *Lasiosina* Becker.. Entomologist's Monthly Magazine.

[B5469794] Evenhuis N. L., Pape T. Systema Dipterorum, Version 2.4. http://www.diptera.dk/.

[B5469803] Ferreira S., Fonseca N., Egeter B., Paupério J., Galhardo M., Oxelfelt F., Aresta S., Martins FMS, Archer J., Corley M., Penado A., Pina S., Jarman S., Beja P. (2018). Deliverable 4.2 (D4.2): Protocol for building and organising reference collections of DNA sequences, EnvMetaGen Project (Grant Agreement No 668981).

[B5471821] Folmer O. M., Black M., Hoeh Wr, Lutz R., Vrijenhoek R. (1994). DNA primers for amplification of mitochondrial cytochrome c oxidase subunit I from diverse metazoan invertebrates. Molecular Marine Biology and Biotechnology.

[B5465289] Hebert Paul D. N., Cywinska Alina, Ball Shelley L., deWaard Jeremy R. (2003). Biological identifications through DNA barcodes. Proceedings of the Royal Society of London. Series B: Biological Sciences.

[B5465299] Kekkonen Mari, Hebert Paul D. N. (2014). DNA barcode-based delineation of putative species: efficient start for taxonomic workflows. Molecular Ecology Resources.

[B5526350] Kress W. John, Wurdack Kenneth J., Zimmer Elizabeth A., Weigt Lee A., Janzen Daniel H. (2005). Use of DNA barcodes to identify flowering plants. Proceedings of the National Academy of Sciences.

[B5472007] Mata V. A., Amorim F., Corley M. F.V., McCracken G. F., Rebelo H., Beja P. (2016). Female dietary bias towards large migratory moths in the European free-tailed bat (*Тadarida teniotis*).. Biology Letters.

[B5471852] Merritt R. W., Courtney G. W., Keiper J. B., Resh V. H., Cardé R. T. (2009). Diptera: (Flies, Mosquitoes, Midges, Gnats). Encyclopedia of Insects.

[B5471868] Pape T., Bickel D., Meier R. (2009). Diptera diversity: Status, challenges and tools..

[B5471877] Pollet M., Andrade R., Gonçalves A., Andrade P., Jacinto V., Almeida J., De Braekeleer A., Van Calster H, Brosens D (2019). Dipterological surveys in Portugal unveil 200 species of long-legged flies, with over 170 new to the country (Diptera: Dolichopodidae). Zootaxa.

[B5471892] Shokralla S., Porter T. M., Gibson J. F., Dobosz R., Janzen D. H., Hallwachs W., Golding G. B., Hajibabaei M. (2015). Massively parallel multiplex DNA sequencing for specimen identification using an Illumina MiSeq platform. Scientific Reports.

[B5472019] Silva L. P, Mata V. A, Lopes P, Pereira P., Jarman S. N., Lopes R. J., Beja P. (2019). Advancing the integration of multi-marker metabarcoding data in dietary analysis of trophic generalists. Molecular Ecology Resources.

[B5471842] Skuhravá Marcela, Martinez Michel, Roques Alain (2010). Diptera. Chapter 10. BioRisk.

[B5471906] Weigand H., Beermann A. J., Ciampor F., Costa F. O, Csabai Z., Duarte S., Geiger M. F., M. Grabowski, Rimet F., Rulik B, Strand M., Szucsich N., Weigand A., Willassen E., Wyler S., Bouchez A, Borja A., Čiamporová-Zaťovičová Z., Ferreira S., Dijkstra K. D., Eisendle U., Freyhof J., Gadawski P., Graf W., Haegerbaeumer A., Van Der Hoorn B. B., Japoshvili B., Keresztes L., Keskin E., Leese F., Macher J., Mamos T., Paz G., Pešić V., Pfannkuchen D. M., Pfannkuchen M. A., Price B. W., Rinkevich B, Teixeira M. A. L., Várbíró G., Ekrem T. (2019). DNA barcode reference libraries for the monitoring of aquatic biota in Europe: Gap-analysis and recommendations for future work. Science of the Total Environment.

[B5471963] Weisser W. W., Siemann E. (2004). Insects and ecosystem function..

